# Tantalum pentoxide: a new material platform for high-performance dielectric metasurface optics in the ultraviolet and visible region

**DOI:** 10.1038/s41377-023-01330-z

**Published:** 2024-01-22

**Authors:** Cheng Zhang, Lu Chen, Zhelin Lin, Junyeob Song, Danyan Wang, Moxin Li, Okan Koksal, Zi Wang, Grisha Spektor, David Carlson, Henri J. Lezec, Wenqi Zhu, Scott Papp, Amit Agrawal

**Affiliations:** 1 School of Optical and Electronic Information & Wuhan National Laboratory for Optoelectronics, Huazhong University of Science and Technology, Wuhan, Hubei 430074 China; 2 National Institute of Standards and Technology, Gaithersburg, MD 20899 USA; 3 University of Maryland, College Park, MD 20742 USA; 4 National Institute of Standards and Technology, Boulder, CO 80305 USA

**Keywords:** Integrated optics, Metamaterials

## Abstract

Dielectric metasurfaces, composed of planar arrays of subwavelength dielectric structures that collectively mimic the operation of conventional bulk optical elements, have revolutionized the field of optics by their potential in constructing high-efficiency and multi-functional optoelectronic systems on chip. The performance of a dielectric metasurface is largely determined by its constituent material, which is highly desired to have a high refractive index, low optical loss and wide bandgap, and at the same time, be fabrication friendly. Here, we present a new material platform based on tantalum pentoxide (Ta_2_O_5_) for implementing high-performance dielectric metasurface optics over the ultraviolet and visible spectral region. This wide-bandgap dielectric, exhibiting a high refractive index exceeding 2.1 and negligible extinction coefficient across a broad spectrum, can be easily deposited over large areas with good quality using straightforward physical vapor deposition, and patterned into high-aspect-ratio subwavelength nanostructures through commonly-available fluorine-gas-based reactive ion etching. We implement a series of high-efficiency ultraviolet and visible metasurfaces with representative light-field modulation functionalities including polarization-independent high-numerical-aperture lensing, spin-selective hologram projection, and vivid structural color generation, and the devices exhibit operational efficiencies up to 80%. Our work overcomes limitations faced by scalability of commonly-employed metasurface dielectrics and their operation into the visible and ultraviolet spectral range, and provides a novel route towards realization of high-performance, robust and foundry-manufacturable metasurface optics.

## Introduction

Recent years have witnessed rapid development in the field of all-dielectric metasurfaces, characterized by high operational efficiency and ease of transmission-mode operation, for spatiotemporal shaping of optical fields in a compact and integration-friendly platform^1–10^. Researchers have demonstrated a series of high-performance metasurface devices operating all the way from the terahertz up to the visible and ultraviolet (UV) frequencies using a range of dielectric materials with different bandgaps^11–16^. These devices have found diverse applications in imaging^17–20^, displaying^21–24^, sensing^25–27^, quantum optics^28–30^, and more. For metasurfaces operating from the near-infrared to the terahertz region, silicon (Si) has been routinely used thanks to the mature and widely-accessible nanofabrication technologies enabled by the Complementary Metal-Oxide-Semiconductor (CMOS) processes built around Si^31–33^. For devices operating in the visible, silicon nitride (SiN_x_)^34–36^, gallium nitride (GaN)^37,38^, and titanium dioxide (TiO_2_)^39–41^ have been employed. High-aspect-ratio subwavelength nanostructures of these materials are fabricated by either a dedicated reactive ion etch (RIE) process, or a resist-based Damascene process incorporating low-temperature atomic layer deposition (ALD). For metasurface optics operating in the UV, wide-bandgap dielectrics, such as hafnium oxide (HfO_2_), niobium pentoxide (Nb_2_O_5_), and aluminum nitride (AlN), are the common materials of choice. Recently, ALD-based Damascene processes have been further developed to implement high-efficiency UV metasurfaces made of hafnium oxide (HfO_2_)^42^ or niobium pentoxide (Nb_2_O_5_)^43^, to overcome limitations of RIE recipes employed for patterning these wide-bandgap dielectrics. AlN has been proposed for UV metasurfaces in numerical studies^44^ and experimental demonstrations using micron-scale zone-plate-like structures^45^ – however, its potential as a viable platform for UV metasurface optics remains to be exploited, as additional investigation is required to pattern AlN into high-aspect-ratio, subwavelength nanostructures with well-controlled sidewall profiles.

In addition to wide-bandgap dielectrics, several alternative material platforms have also been proposed for UV meta-optics. Examples include zirconium dioxide (ZrO_2_) nanoparticle embedded UV-curable resin^46^ and Van der Waals materials^47–50^. By dispersing ZrO_2_ nanoparticles in a UV-curable resin through a proper chemical synthesis process, researchers are able to obtain a new type of imprint resist with a wide bandgap and high refractive index over a broad UV range. Van der Waals materials, such as boron nitride (BN), exhibit high refractive index and broadband optical transparency over the visible and ultraviolet regions, and have been exploited for constructing different excitonic, nanophotonic, and quantum devices. Unfortunately, the materials’ distinctive flake-like morphology might impose severe limitations on their applicability for large-scale devices. Furthermore, their pronounced anisotropic optical responses, while offering an additional dimension for device design, may also introduce challenges within the context of typical metasurface applications. Implementing metasurfaces operating over even shorter wavelength regions that lack materials for transmissive optics, such as vacuum UV (VUV) and extreme UV (EUV), is challenging and an active field of study. Demonstrated endeavors include nonlinear signal generation through dielectric metasurfaces^51,52^, photon nanosieves in opaque metallic films^53^, and vacuum guiding by holes in a silicon membrane^54^.

Realization of efficient dielectric metasurfaces involves shaping high-refractive-index and low-loss dielectric materials into high-aspect-ratio subwavelength nanostructures with well controlled shape, size, and orientation. The performance of a dielectric metasurface is largely determined by its constituent material. An ideal material should have a high refractive index (e.g., $n$ > 2.0) and large bandgap (${E}_{g}$) that facilitate lossless operation at short wavelengths, and at the same time, be patternable into high-aspect-ratio nanostructures using standard CMOS processes with straight and smooth sidewall profiles that guarantee accurate realization of the designed structure. Among commonly employed dielectric materials for metasurfaces, Si can be easily patterned into nanostructures of various geometries, but its narrow bandgap (${E}_{g}$ ≈ 1.1 eV) largely limits operational wavelength down to the near-infrared spectral region. For lossless operation at shorter wavelengths, such as in the visible region, alternative dielectrics such as SiN_x_, GaN and TiO_2_ have been employed. However, their high-quality films are typically grown by certain chemical vapor deposition (CVD) processes using combination of various precursors and the associated nanopatterning techniques are relatively complicated, involving either a carefully developed RIE or Damascene process. For UV and deep-UV regions, wide-bandgap dielectrics such as Nb_2_O_5_ and HfO_2_ are chosen, whose high-aspect-ratio nano-patterning has so far been limited only to the low-temperature-ALD based Damascene process. However, metasurfaces fabricated by ALD-based Damascene processes are not CMOS-compatible and therefore not commercially feasible, largely limiting their scalability. A comparison of different candidate materials is elaborated in Section I, Supplementary Information.

Here, we demonstrate a new dielectric metasurface platform based on tantalum pentoxide (Ta_2_O_5_). We implement high-performance UV and visible metasurface optics through commonly-available physical vapor deposition (PVD) and RIE processes that overcome the reliance of standard dielectric materials on the Damascene process as discussed above. The choice of Ta_2_O_5_, the high-refractive-index component of dielectric mirrors used in the Laser Interferometer Gravitational-Wave Observatory (LIGO)^55^, is justified by (*i*) its wide bandgap ${E}_{g}$ ≈ 4.0 eV (corresponding to a free-space wavelength ${\lambda }_{0}$ = 309 nm) that enables low-loss metasurface operation across the whole visible and near-UV spectrum, and a part of the mid-UV region; and (*ii*) the ability to pattern it into high-aspect-ratio nanostructures using common fluorine-based RIE processes. The large nonlinear optical coefficients of Ta_2_O_5_, which can be further enhanced by doping with other dielectrics such as Nb_2_O_5_^56^, have recently been leveraged for frequency comb generation and optical parametric oscillators in optical micro-resonators^57,58^, and offer a pathway towards the realization of nonlinear metasurfaces for harmonic generation, optical switching and modulation, as well as quantum information processing.

We deposit high-quality, UV- and visible-transparent Ta_2_O_5_ films using a reactive magnetron sputtering process and pattern the films into high-aspect-ratio nanostructures through a fluorine-gas-based RIE process. We implement a series of high-efficiency UV and visible metasurfaces offering a set of representative light-field modulation functionalities. We first show a group of UV metalenses with different numerical apertures (NAs) ranging from 0.5 to 0.7, which are made of Ta_2_O_5_ nanopillars of circular in-plane cross-sections and exhibit polarization-insensitive focusing capability down to the diffraction limit. The metalenses demonstrate experimentally measured focusing efficiencies up to ≈65% at an operational wavelength of 325 nm. We then realize broadband UV and visible meta-holograms, which are made of Ta_2_O_5_ nanopillars of elliptical in-plane cross-sections and provide spin-selective holographic projection across the near-UV and blue region. The devices exhibit peak operational efficiencies exceeding 70% at 325 nm. We further demonstrate a series of structural color generating metasurfaces made of resonant Ta_2_O_5_ nanopillars supporting geometrical Mie resonances. The devices produce bright and high-purity reflection colors across the entire visible region, with measured peak efficiencies of ≈80%. Our work provides a novel route towards robust and low-cost fabrication of high performance dielectric metasurfaces operating in the UV and visible regions using CMOS compatible processes, and promotes realization of compact-form-factor and multifunctional photonic systems based on flat optical elements in these critical spectral regions.

## Results

### Material preparation and nanostructure fabrication

**Fig. 1:**
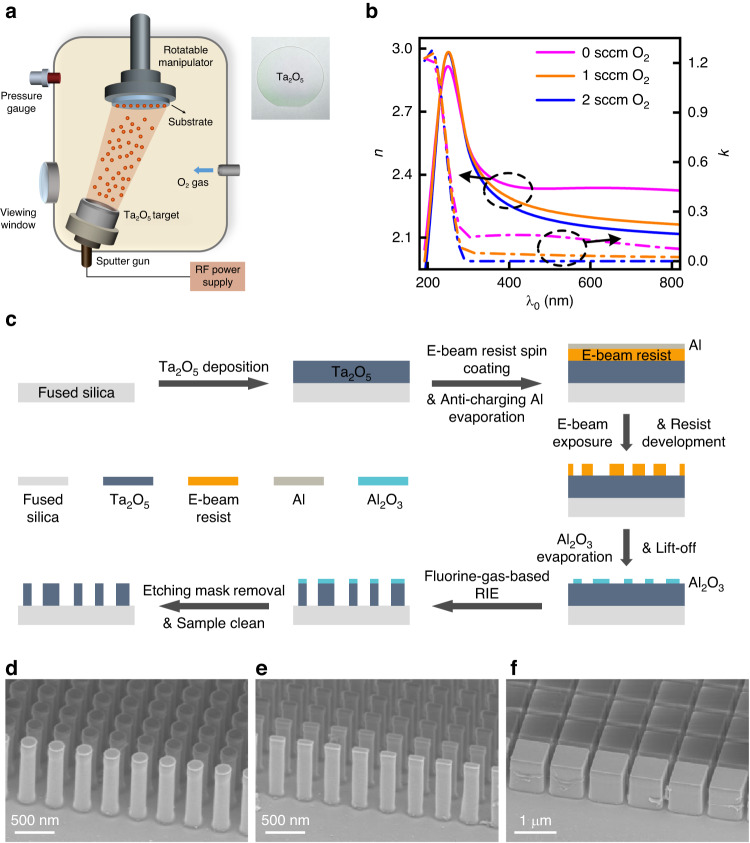
Material preparation and nanostructure fabrication. **a** Schematic representation of the O_2_-gas-based RF reactive magnetron sputtering process for Ta_2_O_5_ film deposition. Inset: Photo of a 400-nm-thick Ta_2_O_5_ film deposited on a 50-mm-diameter fused silica substrate. **b** Refractive index *n* and extinction coefficient $\kappa$ of the sputter-deposited Ta_2_O_5_ films with different O_2_ gas flow rates, characterized by spectroscopic ellipsometry. **c** Fabrication flow chart of the Ta_2_O_5_ metasurface optics, whose key steps include film deposition using the developed sputtering recipe, electron beam lithography, Al_2_O_3_ etching mask lift-off, and fluorine-gas-based RIE. **d**–**f** Scanning electron micrographs (SEMs) of details of fabricated Ta_2_O_5_ nanopillars with different geometric shapes

Ta_2_O_5_ films are deposited through a radiofrequency (RF) magnetron sputtering process using a Ta_2_O_5_ target (Fig. 1a). Inset shows the picture of a 400-nm-thick Ta_2_O_5_ film deposited on a 50-mm-diameter fused silica substrate. Film thickness and refractive index are both characterized by spectroscopic ellipsometry, as detailed in the Materials and Methods section. Although Ta_2_O_5_ has an intrinsic wide bandgap, films deposited using conventional magnetron sputtering would inevitably have defects, and thus, exhibit sub-bandgap absorption (Fig. 1b, magenta curves). To solve this issue, we develop a reactive RF sputtering process with Oxygen (O_2_) gas. As the flow rate of O_2_ gas increases, the sputtered Ta_2_O_5_ film exhibits a monotonically decreasing absorption coefficient $k$ over the UV and visible regions (Fig. 1b, orange and blue curves). With an O_2_ flow rate of 2 standard cubic centimeters per minute (sccm), the deposited Ta_2_O_5_ film exhibits a refractive index $n$ > 2.27 over the entire UV range, as well as negligible absorption coefficient $k$ down to ${\lambda }_{0}$ ≈ 300 nm. Further increase of the O_2_ flow rate does not lead to any noticeable change in the refractive indices of the obtained films. It is worth noting that our developed film deposition process does not require any special substrate (e.g., for lattice matching), or heating (cooling) of the substrate during film deposition, therefore enabling a straightforward and high-throughput deposition of high optical quality Ta_2_O_5_ films onto various types of substrates at room temperature. Although Ta_2_O_5_ films are deposited using magnetron sputtering process in this study, other PVD processes (e.g., thermal evaporation, electron beam evaporation, pulsed laser deposition, etc.) as well as ALD can be utilized for high-quality Ta_2_O_5_ film preparation.

The metasurface optics fabrication (Fig. 1c) starts with depositing Ta_2_O_5_ film of target thickness onto a 500-µm-thick fused silica substrate using the developed reactive sputtering process. Then, a 200-nm-thick layer of positive electron beam resist is spin-coated onto the film, followed by evaporating a 20-nm-thick anti-charging aluminum (Al) layer. A 100 keV electron beam lithography system is used to expose the nanostructure patterns, followed by Al layer removal with diluted tetramethylammonium hydroxide (TMAH) and resist development with hexyl acetate at 4 °C. The developed patterns in the resist layer are transferred to an evaporated 50-nm-thick aluminum oxide (Al_2_O_3_) layer through a lift-off process. Using the nanostructured Al_2_O_3_ layer as the etch mask, inductively coupled-plasma reactive ion etching (ICP-RIE, gas mixture: C_4_F_8_ and O_2_; ICP power: 2000 W; RF power: 15 W) is performed to pattern the underlying Ta_2_O_5_ layer at 50 °C and create high-aspect-ratio Ta_2_O_5_ nanopillars. The metasurface optics fabrication concludes with soaking the sample in a mixture of hydroperoxide and ammonia hydroxide solutions heated at 80 °C for 30 minutes, to remove the Al_2_O_3_ etch mask and any etch residue.

Representative scanning electron micrographs (SEMs) of fabricated Ta_2_O_5_ nanopillars are displayed in Fig. 1d to f, showing straight and smooth sidewall profiles. Patterns with different geometric shapes and sizes, frequently used in a plethora of dielectric metasurface devices, are chosen to illustrate the versatility of the developed fabrication process. In addition, Fig. 1d and e correspond to etching situations with large open (exposed) areas, while Fig. 1f corresponds to situations with small open areas. Figure 1d shows a group of high-aspect-ratio (≈10:1) Ta_2_O_5_ nanopillars with circular (symmetric) in-plane cross-sections, which are frequently used to construct dielectric metasurfaces with polarization-independent response. Figure 1e shows an array of Ta_2_O_5_ nanopillars with an even higher aspect ratio (≈12:1), which exhibit rectangular (asymmetric) in-plane cross-sections. Different from the symmetric Ta_2_O_5_ nanopillars, these structures are typically employed for metasurfaces with polarization-dependent (e.g., linear-polarization-multiplexed, spin-multiplexed, spin-selective, etc.) responses. Figure 1f shows an array of closely packed Ta_2_O_5_ nanopillars with a moderate aspect ratio. These structures can be used to create dielectric Mie resonators for spectral filtering, local optical field enhancement, nonlinear harmonic generation, etc.

### Polarization-independent UV metalenses

To demonstrate the versatility of this material platform and the developed nanopatterning technique, we choose to implement two different categories of metasurfaces for UV light wavefront shaping respectively based on propagation phase^31,59^ and geometric phase^39,60^, which are the two most representative wavefront control methods currently employed by dielectric metasurfaces.

**Fig. 2:**
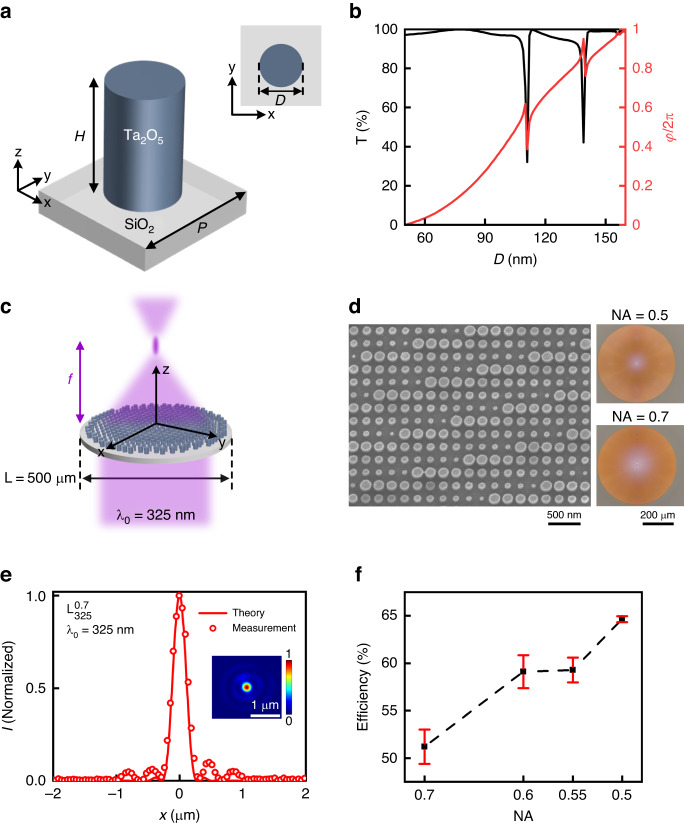
Polarization-independent UV metalenses. **a** Schematic representation of a polarization-independent metasurface unit cell, consisting of a high-aspect-ratio Ta_2_O_5_ circular pillar of height *H* and cross-sectional diameter *D*, arranged on a SiO_2_ substrate to form a square lattice with subwavelength lattice spacing *P*. **b** Transmission intensity *T* and phase shift *φ* for an incident light of free-space wavelength ${\lambda }_{0}$ = 325 nm, as a function of pillar diameter *D*. A corresponding pillar height (*H* = 340 nm), lattice spacing (*P* = 200 nm), and a range of pillar diameters (*D* $\in$ [50, 160] nm) are chosen. For ease of display, the phase shift for pillar of diameter *D* = 50 nm is set to zero. **c** Schematic representation of focusing by a polarization-independent, 500-µm-diameter metalens under normal-incidence, plane-wave illumination at ${\lambda }_{0}$ = 325 nm. **d** Left panel: Top-view SEM image of a fabricated NA = 0.5 metalens ${{\rm{L}}}_{325}^{0.5}$, consisting of cylindrical Ta_2_O_5_ nanopillars of varying diameters. Right panel: Optical micrographs of a NA = 0.5 metalens (${{\rm{L}}}_{325}^{0.5}$, upper part) and a NA = 0.7 metalens (${{\rm{L}}}_{325}^{0.7}$, lower part). **e** Intensity distribution at the focal plane along the x-axis (dots), measured for metalens ${{\rm{L}}}_{325}^{0.7}$. Theoretical prediction (line) is shown for comparison. Inset: Intensity distribution at the focal plane. **f** Measured focusing efficiency as a function of metalens numerical aperture (NA). Error bars denote two standard deviations of the measured data

The propagation-phase-based metasurface consists of a square lattice of Ta_2_O_5_ cylindrical nanopillars, where the diameter of each pillar varies as a function of its spatial position on the metasurface plane (Fig. 2a). Each nanopillar acts as a truncated dielectric waveguide with top and bottom interfaces of low reflectivity, through which light propagates with a transmittance *T* and phase shift $\varphi$ controlled by the pillar height *H*, pillar diameter *D*, and lattice spacing *P*. Due to the symmetric nature of the nanopillar’s in-plane cross-section, propagation phase is independent of incident light polarization. To design the metasurface, the transmittance, *T*, and induced phase shift, $\varphi ,$ of an array of cylindrical Ta_2_O_5_ pillars of diameter *D*, height *H*, and lattice spacing *P*, under plane-wave normal incidence illumination at the target operational free-space wavelength ${\lambda }_{0}$ = 325 nm, are computed using finite-difference-time-domain (FDTD) simulations with periodic boundary conditions. After several rounds of iteration, a pillar height (*H* = 340 nm) and subwavelength lattice spacing (*P* = 200 nm) is chosen, along with a range of pillar diameters (*D* $\in$ [50, 160] nm) that yield phase shifts varying over a full range from 0 to 2π, while maintaining a relatively high and constant transmittance for incident light at ${\lambda }_{0}$ = 325 nm (Fig. 2b).

Using the obtained nanopillar library, we implement a series of metalenses with numerical aperture (NA) values ranging from 0.5 to 0.7. Four 500-µm-diameter metalenses, ${{\rm{L}}}_{325}^{0.5}$, ${{\rm{L}}}_{325}^{0.55}$, ${{\rm{L}}}_{325}^{0.6}$, and ${{\rm{L}}}_{325}^{0.7}$, are all designed to focus UV light of ${\lambda }_{0}\,$= 325 nm (Fig. 2c), but with different NA values of 0.5, 0.55, 0.6, and 0.7, respectively. The associated focal lengths, ${{\rm{f}}}_{325}^{0.5}$, ${{\rm{f}}}_{325}^{0.55}$, ${{\rm{f}}}_{325}^{0.6}$, and ${{\rm{f}}}_{325}^{0.7}$, are 433 µm, 379.6 µm, 333.3 µm, and 255 µm, respectively. Singlet-mode focusing of a normally-incident plane wave can be achieved by implementing a radially-symmetric phase shift function ${\varphi }^{L}\left(x,y,{\lambda }_{0}\right)=\mathrm{mod}\left((2\pi /{\lambda }_{0})\,\left(f-\sqrt{{x}^{2}+{y}^{2}+{f}^{2}}\right),2\pi \right)$ over the metasurface plane. Here, $f$ is the focal length, *x* and *y* are in-plane distances along orthogonal directions from the centre of the lens, given incident light propagating along the *z*-direction.

The left panel of Fig. 2d shows the SEM image of the details of a fabricated NA = 0.5 metalens ${{\rm{L}}}_{325}^{0.5}$, consisting of cylindrical nanopillars with spatially varying diameters. The right panel of Fig. 2d displays the optical micrographs of a NA = 0.5 metalens (${{\rm{L}}}_{325}^{0.5}$, upper part) and a NA = 0.7 metalens (${{\rm{L}}}_{325}^{0.7}$, lower part). The metalenses are characterized using a custom-built optical setup (detailed in the Materials and Methods section). For each metalens, the measured intensity distribution at the device’s focal plane reveals a circularly-symmetric focal spot, characterized by a cross section that closely matches the intensity distribution theoretically predicted for a diffraction-limited lens with certain NA, given by the Airy disk function $I\left(x\right)={\left[2{J}_{1}(A)/A\right]}^{2}$, where ${J}_{1}$ is the first-order Bessel function of the first kind, and $A=2\pi ({\rm{NA}})x/{\lambda }_{0}$ (Fig. 2e and Fig. S2, Supplementary Information). The focusing efficiencies, defined as the ratio of the optical power of the focused spot to the total power illuminating the metalens, are measured to be (64.7 ± 0.3)% (${{\rm{L}}}_{325}^{0.5}$), (59.3 ± 1.3)% (${{\rm{L}}}_{325}^{0.55}$), (59.1 ± 1.7)% (${{\rm{L}}}_{325}^{0.6}$), and (51.2 ± 1.8)% (${{\rm{L}}}_{325}^{0.7}$). The cited uncertainties represent two standard deviations of the measured data. The metalens efficiency exhibits a slow and monotonical decrease as the device’s NA increases (Fig. 2f). These obtained efficiencies are comparable to those of HfO_2_-based UV metalenses, which are fabricated through a resist-based Damascene process^42^.

### Spin-selective and broadband metaholograms

**Fig. 3:**
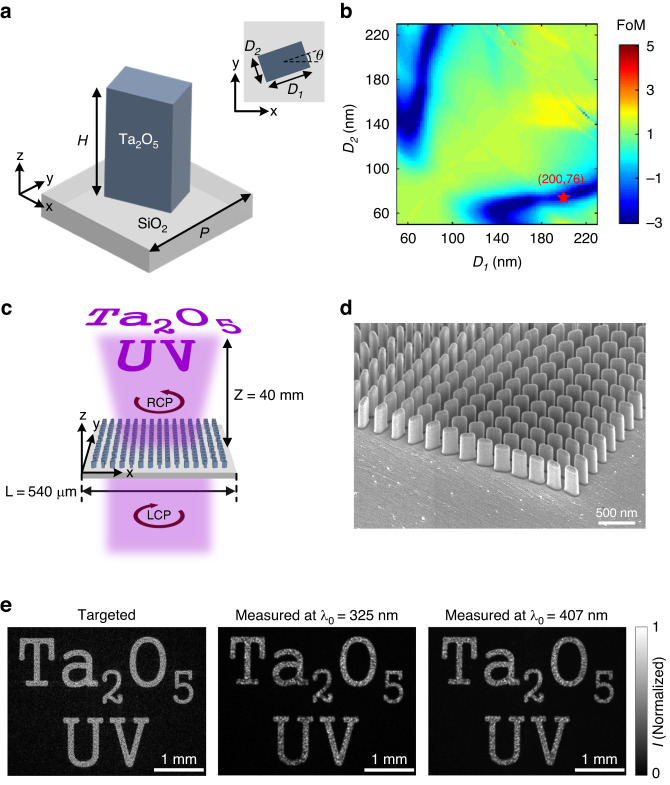
Spin-selective and broadband metaholograms. **a** Schematic representation of a spin-selective metasurface unit cell, consisting of a high-aspect-ratio Ta_2_O_5_ cylindrical pillar of height *H*, with an elliptical cross-section (major and minor axis lengths of *D*_1_ and *D*_2_, respectively), and rotation angle $\theta$ arranged on a SiO_2_ substrate to form a square lattice with subwavelength lattice spacing *P*. Spin-selective optical field modulation can be implemented via the variation of *D*_1_, *D*_2_, and $\theta$ as a function of nanopillar position within the lattice. **b** Half-waveplate figure of merit (FoM) versus nanopillar in-plane dimensions (*D*_1_ and *D*_2_) at target free-space wavelength ${\lambda }_{0}$ = 325 nm, where the blue-colored regions (i.e., low-FoM value regions) correspond to various combinations of *D*_1_ and *D*_2_ that satisfy the half-waveplate-like operation. Pillar height *H* = 380 nm and lattice spacing *P* = 270 nm are used during the calculation. The chosen pillar geometry in this study (*D*_1_ = 200 nm and *D*_2_ = 76 nm) is denoted by a red star. **c** Schematic representation of the holographic image projection by a spin-selective metahologram, ${{\rm{H}}}_{325}^{{\rm{LCP}}}$, under normal-incidence, left-handed circularly polarized (LCP) plane-wave illumination at ${\lambda }_{0}$ = 325 nm. **d** SEM of details of a fabricated metahologram ${{\rm{H}}}_{325}^{{\rm{LCP}}}$, consisting of elliptical Ta_2_O_5_ nanopillars of varying rotation angles. Viewing angle: 52°. **e** Targeted (left panel) and measured (middle and right panels) holographic images projected by the metahologram ${{\rm{H}}}_{325}^{{\rm{LCP}}}$ in the *z* = 40 mm plane. The targeted image is numerically computed assuming an ideal metahologram realization with both the designed phase shift profile ${\varphi }_{325}^{H}(x,y,{\lambda }_{0})$ and unity transmittance T, under normal-incidence illumination at ${\lambda }_{0}$ = 325 nm

We also implement geometric-phase-based metasurfaces for spin-selective hologram projection, which operate over a broad UV and visible spectrum under left-handed circularly polarized (LCP) light illumination. The geometric-phase-based metasurface consists of a square lattice of Ta_2_O_5_ elliptical nanopillars shaped identically, but with spatially-varying rotation angles (Fig. 3a). Each nanopillar acts as a miniaturized half-waveplate for incident circularly-polarized light, and transmits with circular polarization of opposite handedness along with a phase delay that equals twice the pillars’ rotation angle. Such phase modulation is typically referred to as the Pancharatnam-Berry (PB) phase or geometric phase^60^. To design the geometric-phase-based metasurface operating at ${\lambda }_{0}$ = 325 nm, the transmittance and phase shift for propagation of 325 nm wavelength light, linearly-polarized either (*i*) parallel to the major axis (${{\rm{T}}}_{1}$ and ${\Delta }_{1}$), or (*ii*) parallel to the minor axis (${{\rm{T}}}_{2}$ and ${\Delta }_{2})$ of an array of elliptical Ta_2_O_5_ nanopillars are computed using FDTD simulations with periodic boundary conditions. For a chosen pillar height *H* = 380 nm and lattice spacing *P* = 270 nm, the major and minor axis lengths of the pillar, ${D}_{1}$ and ${D}_{2}$, are iteratively varied to identify orthogonal principal axis combinations simultaneously leading to |${\Delta }_{1}$ − ${\Delta }_{2}$|≈π and ${{\rm{T}}}_{1}$ ≈ ${{\rm{T}}}_{2}$, in other words, half-waveplate-like operation. To facilitate the above parameter search process, a figure-of-merit (FoM) function is defined as ${\rm{FoM}}={\log }_{10}\left(\left|\frac{{T}_{1}}{{T}_{2}}{e}^{\left(i\left({\Delta }_{1}-{\Delta }_{2}\right)\right)}-{e}^{i\pi }\right|\right)$ and displayed in Fig. 3b, where the blue-colored regions (i.e., low-FoM value regions) correspond to various combinations of ${D}_{1}$ and ${D}_{2}$ that satisfy the target half-waveplate operation. The chosen pillar geometry in this study (${D}_{1}\,$ = 200 nm and ${D}_{2}\,$ = 76 nm) is denoted by a red star.

The implemented metahologram (denoted ${{\rm{H}}}_{325}^{{\rm{LCP}}}$), which occupies a square area with a side length of 540 µm, is mapped to a Cartesian coordinate system where the constituent metasurface is in the $z$ = 0 plane and the 1st *x-y* quadrant, with one corner positioned at the origin (Fig. 3c). The metahologram operates by converting a left-handed circularly polarized (LCP) incident light into a right-handed circularly polarized (RCP) light upon transmission, and at the same time, imparting a spatially-varying phase shift modulation onto the transmitted light for the subsequent holographic image formation. The Gerchberg-Saxton (GS) algorithm^61^ is employed to calculate the phase shift profiles, ${\varphi }_{325}^{H}(x,y,{\lambda }_{0})$, required to project a holographic “Ta_2_O_5_ UV” image (4 mm in width) located in the $z$ = 40 mm plane, under normally-incident plane-wave illumination of ${\lambda }_{0}$ = 325 nm (Fig. S3, Supplementary Information). An additional offset of $y$ = $-\,$3 mm is added to avoid overlap of the generated holographic image with the residual directly transmitted beam.

**Fig. 4:**
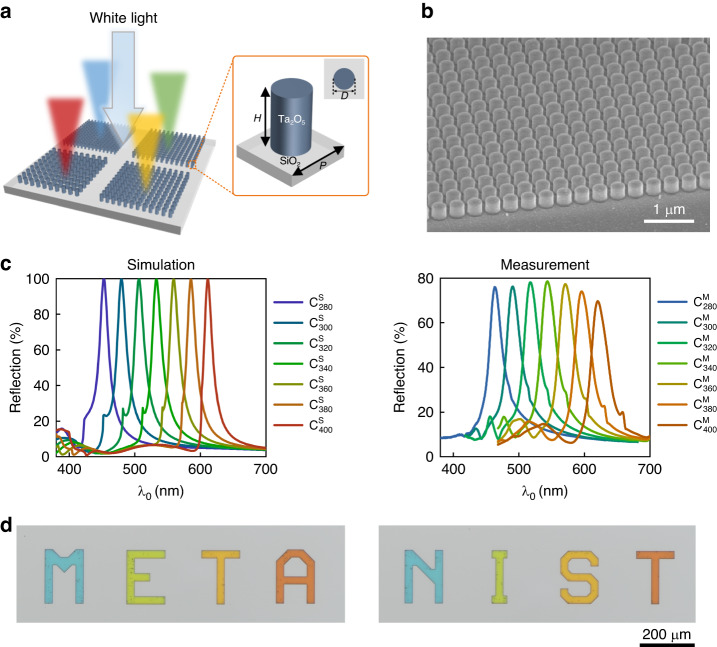
Structural color generating metasurfaces. **a** Schematic representation of the reflection-mode structural color generation by an array of Ta_2_O_5_ Mie resonators, under normal-incidence white light. Inset: Schematic illustration of a Ta_2_O_5_ Mie resonator unit cell, consisting of a Ta_2_O_5_ circular pillar of height *H* and cross-sectional diameter *D*, arranged on a SiO_2_ substrate to form a square lattice with subwavelength lattice spacing *P*. By fixing the pillar height (*H*) as 240 nm and filling ratio (*f* = *D*/*P*) as 0.7 and varying the pillar lattice spacing (*P*) from 260 nm to 400 nm, different reflection colors covering the full visible range are created. **b** SEM image of a fabricated structural color generating metasurface of lattice spacing of 300 nm (C_300_), consisting of cylindrical Ta_2_O_5_ nanopillars with circular cross-sections, of uniform sizes and straight side-wall profiles. Viewing angle: 54°. **c** Simulated (left panel) and measured (right panel) reflection spectra of seven structural color generating metasurfaces with different lattice spacings. For each curve, the employed color is deliberately chosen according to the standard RGB values calculated from the associated reflection spectrum and indicates the perceived color by human eye. **d** Optical micrographs of colored words “META” and “NIST” printed by the Ta_2_O_5_ metasurfaces

The SEM image of the fabricated metahologram ${{\rm{H}}}_{325}^{{\rm{LCP}}}$ is displayed in Fig. 3d. The sample is characterized using a custom-built imaging system (detailed in the Materials and Methods section). The measured image projected by the device under ${\lambda }_{0}$ = 325 nm illumination is displayed in the middle panel of Fig. 3e, which faithfully replicates the shape of the corresponding target image (left panel, Fig. 3e) numerically computed assuming an ideal metahologram realization with both the designed phase shift profile ${\varphi }_{325}^{H}(x,y,{\lambda }_{0})$ and unity transmittance T. The measured efficiency, defined as the ratio of the total optical power of the holographic image to the total power illuminating the structure, is (75.9 ± 1.1)%. The cited uncertainty represents two standard deviations of the measured data. The obtained efficiency value is comparable to those of recently reported TiO_2_-based metaholograms operating in the visible^39^, and HfO_2_-based devices operating in the UV^42^. The broadband characteristic of the employed geometric phase and absorption-free nature of Ta_2_O_5_ across the UV and visible regions, allow the metahologram to operate over a wide wavelength range. We experimentally characterize device performance under an LCP laser beam illumination at ${\lambda }_{0}$ = 407 nm. The captured holographic image (right panel, Fig. 3e) closely resembles the target image displayed in the left panel of Fig. 4e, with a measured efficiency of (37.3 ± 0.3)%.

### Structural color generating metasurfaces

The deposited Ta_2_O_5_ films exhibit a high refractive index and negligible extinction coefficient at wavelengths longer than 300 nm, the material’s bandgap. As observed in Fig. 1b, the 2 sccm O_2_-deposited Ta_2_O_5_ is free of optical absorption and simultaneously displays a refractive index $n$ > 2.12 over the whole visible range (380 nm to 750 nm). This offers a promising material platform for realizing high-performance metasurface devices operating in the visible spectrum, thus far achieved using other materials such as TiO_2_, SiN_x_ and GaN.

We implement structural color generation based on Ta_2_O_5_ Mie resonators as a demonstration of high-performance metasurfaces at visible wavelengths. Figure 4a depicts the schematic of the Ta_2_O_5_-based structural color generating metasurface, which consists of a square lattice of Ta_2_O_5_ cylindrical nanopillars with height $H$ and diameter $D$, arrayed over a lattice spacing *P* on fused silica substrate. Mie resonances^62–64^ are supported by the high-refractive-index Ta_2_O_5_ nanopillars, generating distinct spectral peaks in reflection. The central wavelength of the reflection peak (i.e., the generated reflection color) can be adjusted by varying one or several geometric parameters of the nanopillar, such as its height, diameter, or lattice spacing. In our design, we fix the pillar height ($H$) as 240 nm, and the pillar filling ratio ($f=D/P$) as 0.7. By varying the lattice spacing ($P$) from 260 nm to 400 nm, different reflection colors covering the full visible range are created (Left panel, Fig. 4c). Moreover, the generated colors exhibit narrow spectral widths (indicating high color purity), and at the same time, peak reflection intensities of up to 100% (indicating high color brightness). In the figure, the employed color for each curve is deliberately chosen according to standard RGB (sRGB) values calculated from the associated reflection spectrum and indicates colors perceived by the human eye. Angular response of the designed color generating metasurface and ways to tune its spectral linewidth are respectively elaborated in Section IV and V, Supplementary Information.

Seven 500-µm-square-area Ta_2_O_5_ metasurfaces (${{\rm{C}}}_{280}$, ${{\rm{C}}}_{300}$, ${{\rm{C}}}_{320}$, ${{\rm{C}}}_{340}$, ${{\rm{C}}}_{360}$, ${{\rm{C}}}_{380}$, and ${{\rm{C}}}_{400}$) of lattice spacing varying from 280 nm to 400 nm with a step size of 20 nm, are fabricated and characterized. A representative SEM image of a 300 nm period sample, ${{\rm{C}}}_{300}$, is displayed in Fig. 4b, showing pillars of uniform sizes and straight side-wall profiles. Measured reflection spectra of the fabricated samples are plotted in the right panel of Fig. 4c. Similarly, the color of each curve is chosen based on the sRGB values calculated from the corresponding measured reflection spectrum. The simulated and measured reflection spectra agree well, in terms of both the line shape and perceived color. For all obtained samples, the peak reflection intensities are close to ≈80%, which is comparable to or even higher than previously demonstrated Si- and TiO_2_-based structural color metasurfaces^22,41,65^. In addition, the full width at half maximum (FWHM) values of the main peaks for all samples are narrower than ≈20 nm, suggesting high purity of the achieved colors. We calculate the color coordinates from both the simulated and measured spectra, and plotted the coordinates in the CIE 1931 XYZ chromaticity diagram (Section VI, Supplementary Information). Furthermore, we implement color printing of words “META” and “NIST” utilizing four different metasurface structures with lattice spacings respectively set as 280 nm, 340 nm, 360 nm, and 400 nm. When the samples are illuminated with a white light under bright-field microscope, images with each constituent letter of uniform and vivid color can be clearly observed (Fig. 4d).

## Discussion

In conclusion, we present a new dielectric material platform based on tantalum pentoxide (Ta_2_O_5_), for realizing high-efficiency metasurface optics over the UV and visible spectrum. Ta_2_O_5_ is characterized by a wide bandgap value of ≈4.0 eV, enabling low-loss metasurface operation across the whole visible and near-UV regions, as well as part of the mid-UV region. Moreover, the material can be easily deposited uniformly onto various substrates over large areas using simple physical vapor deposition and patterned into high-aspect-ratio nanostructures through common fluorine-gas-based reactive ion etching. We demonstrate an array of high-performance UV and visible Ta_2_O_5_ metasurfaces with representative wavefront shaping capabilities, namely, polarization-independent UV metalenses of focusing efficiencies up to 65%, spin-selective metaholograms operating in the near-UV and blue spectrum with peak efficiencies exceeding 75%, and full-visible-region structural color generating Mie resonators exhibiting peak reflection intensities close to 80%. Thanks to this versatile material platform, these devices, though based on conventional designs, already exhibit performance that is comparable to state-of-the-art UV and visible metasurfaces using other dielectric materials. We believe their performance can be further improved by advanced metasurface design strategies, such as topology optimization^66^ and machine learning^67^. Our work offers a new vista for realizing low-loss and fabrication-friendly dielectric metasurfaces operating in the UV and visible regions, enabling various applications such as atom trapping, high-resolution imaging, and advanced display with a compact form factor.

## Materials and Methods

### Film thickness and refractive index characterization

The Ta_2_O_5_ layer is sputter-deposited onto a silicon wafer coated with a 300 nm thick thermal oxide layer. The film’s refractive index and thickness value are characterized by reflection-mode spectroscopic ellipsometry using the interference enhancement method^68,69^, at three different angles of incidence (55°, 65°, and 75°) with respect to the normal to the plane of the Ta_2_O_5_ layer.

### Metalens characterization

To characterize the metalenses, a continuous wave (CW) Helium–Cadmium (HeCd) laser (${\lambda }_{0}$ = 325 nm) is employed to illuminate each sample at normal incidence. The intensity distribution on the lens focal plane is captured using a custom-built imaging system including an NA = 0.75 objective and an electron multiplying charge-coupled device (EMCCD) camera. The system magnification, characterized by translating the focal spot within the field of view of the objective using a high-precision motorized stage, is measured to be ≈546 (for ${{\rm{L}}}_{325}^{0.5}$), ≈548 (for ${{\rm{L}}}_{325}^{0.55}$), ≈550 (for ${{\rm{L}}}_{325}^{0.6}$), and ≈547 (for ${{\rm{L}}}_{325}^{0.7}$). The physical size of the focal spot projected by each metalens is derived based on the calibrated magnification and the pixel size of the EMCCD.

### Metahologram characterization

To characterize the metahologram, a CW HeCd laser (${\lambda }_{0}$ = 325 nm) is employed to illuminate the sample at normal incidence. A pair of linear polarizer and half-waveplate is used to convert the state of polarization of the incident beam to LCP. An EMCCD camera is placed in the plane of the holographic image to directly record the projected holographic image.

### Supplementary information

Supplementary Information

Figure 1

Figure 2

Figure 3

Figure 4

## Data Availability

The data that support the plots within this paper and other finding of this study are available from the corresponding authors upon request.
