# Supplementary material for: Tantalum pentoxide: a new material platform for high-performance dielectric metasurface optics in the ultraviolet and visible region
Source: Light Sci Appl. 2024 Jan 22;13:23. doi: 10.1038/s41377-023-01330-z (PMC10800353; doi:10.1038/s41377-023-01330-z)
Supplement: Supplementary file 1 — Supplementary Information [file 41377_2023_1330_MOESM1_ESM.pdf]

Supplementary Information for

Tantalum Pentoxide: A New Material Platform for High-performance Dielectric Metasurface Optics in the Ultraviolet and Visible Region

Cheng Zhang[1+,*], Lu Chen[2,3+], Zhelin Lin[1], Junyeob Song[2], Danyan Wang[1], Moxin Li[1], Okan Koksal[2], Zi Wang[2,3], Grisha Spektor[4], David Carlson[4], Henri J. Lezec[2], Wenqi Zhu[2,3], Scott Papp[4], and Amit Agrawal[2,*]

[1]School of Optical and Electronic Information & Wuhan National Laboratory for Optoelectronics, Huazhong University of Science and Technology, Wuhan, Hubei 430074, China

[2]National Institute of Standards and Technology, Gaithersburg, MD, 20899, USA

[3]University of Maryland, College Park, MD 20742, USA

[4]National Institute of Standards and Technology, Boulder, CO, 80305, USA

[+] Equal contributors

[*] Email: cheng.zhang@hust.edu.cn; amit.agrawal@nist.gov

I. Comparison between different material platforms for the UV and visible regions

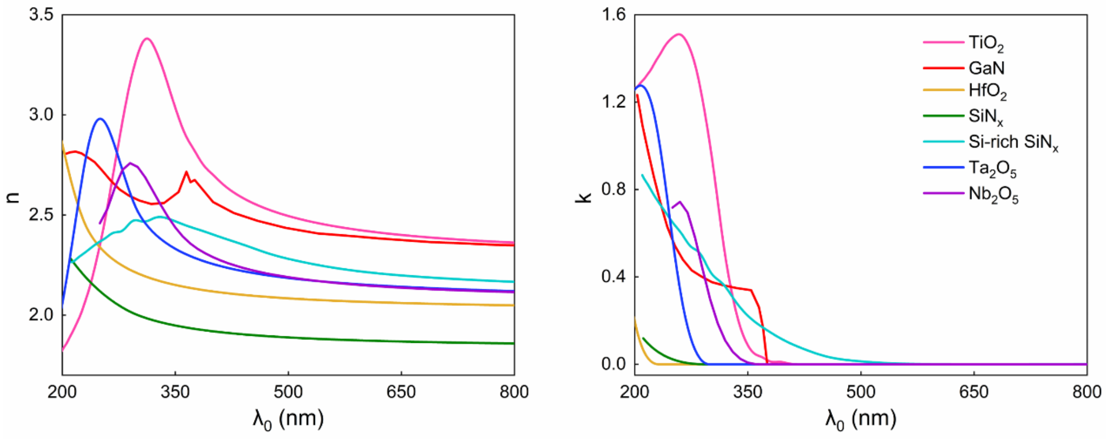

Figure S1: Refractive indices and extinction coefficients of different candidate materials for the UV and visible regions. For SiN_x, its various properties including refractive index, extinction coefficient, bandgap value, and film stress, can be tuned by varying the deposition conditions (e.g., temperature, precursor combination, gas ratio, etc.). Here, the curves for a stoichiometric silicon nitride film and silicon-rich silicon nitride film are plotted as representative examples.

Table S1: Comparison of different candidate materials for the UV and visible regions in terms of typical applicable wavelength region, film deposition and nano-patterning methods.

**Material**	**Typical applicable wavelength region**	**Material deposition**	**Nanostructure patterning**
GaN	$\lambda_0 > 360$ nm (Visible)	MOCVD	RIE
TiO_2	$\lambda_0 > 380$ nm (Visible)	ALD or PVD	Damascene lithography or RIE
SiN_x	$\lambda_0 > 300$ nm (Near-UV to visible)	PECVD or LPCVD	RIE
Nb_2O_5	$\lambda_0 > 345$ nm (Near-UV to visible)	ALD	Damascene lithography
HfO_2	$\lambda_0 > 220$ nm (Deep-UV to visible)	ALD	Damascene lithography
Ta_2O_5	$\lambda_0 > 300$ nm (Mid-UV to visible)	PVD	RIE

II. Measured Cross-focus cuts and intensity distributions for metalenses $\mathbf{L_{325}^{0.5}}$, $\mathbf{L_{325}^{0.55}}$, and $\mathbf{L_{325}^{0.6}}$

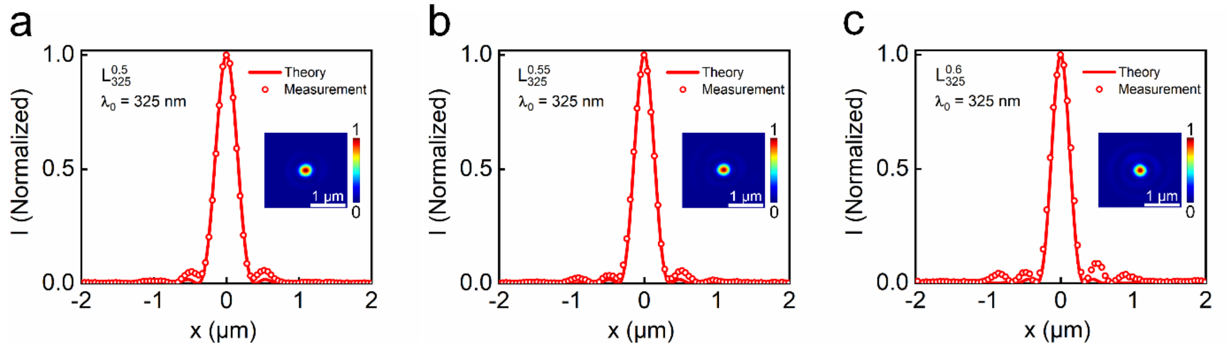

Figure S2: Cross-focus cuts and intensity distributions in the focal plane, respectively measured for metalens $L_{325}^{0.5}$ (a), $L_{325}^{0.55}$ (b), and $L_{325}^{0.6}$ (c). In each figure, the theoretically predicted cross-focus cut is plotted for reference.

III. Designed phase shift profile for the spin-selective metahologram $\mathbf{H_{325}^{LCP}}$

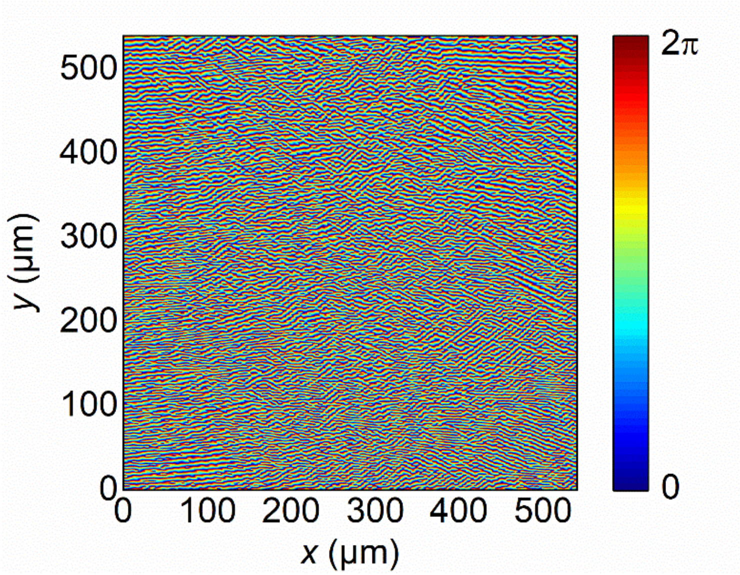

Figure S3: Metasurface phase-shift profile, φ_{325}^{H}, designed to project a holographic "Ta_2O_5 UV" image (4 mm in width) located in the z = 40 mm plane, under normally-incident plane-wave illumination of λ_0 = 325 nm.

IV. Angular response of the Ta_2O_5 structural color generating metasurfaces

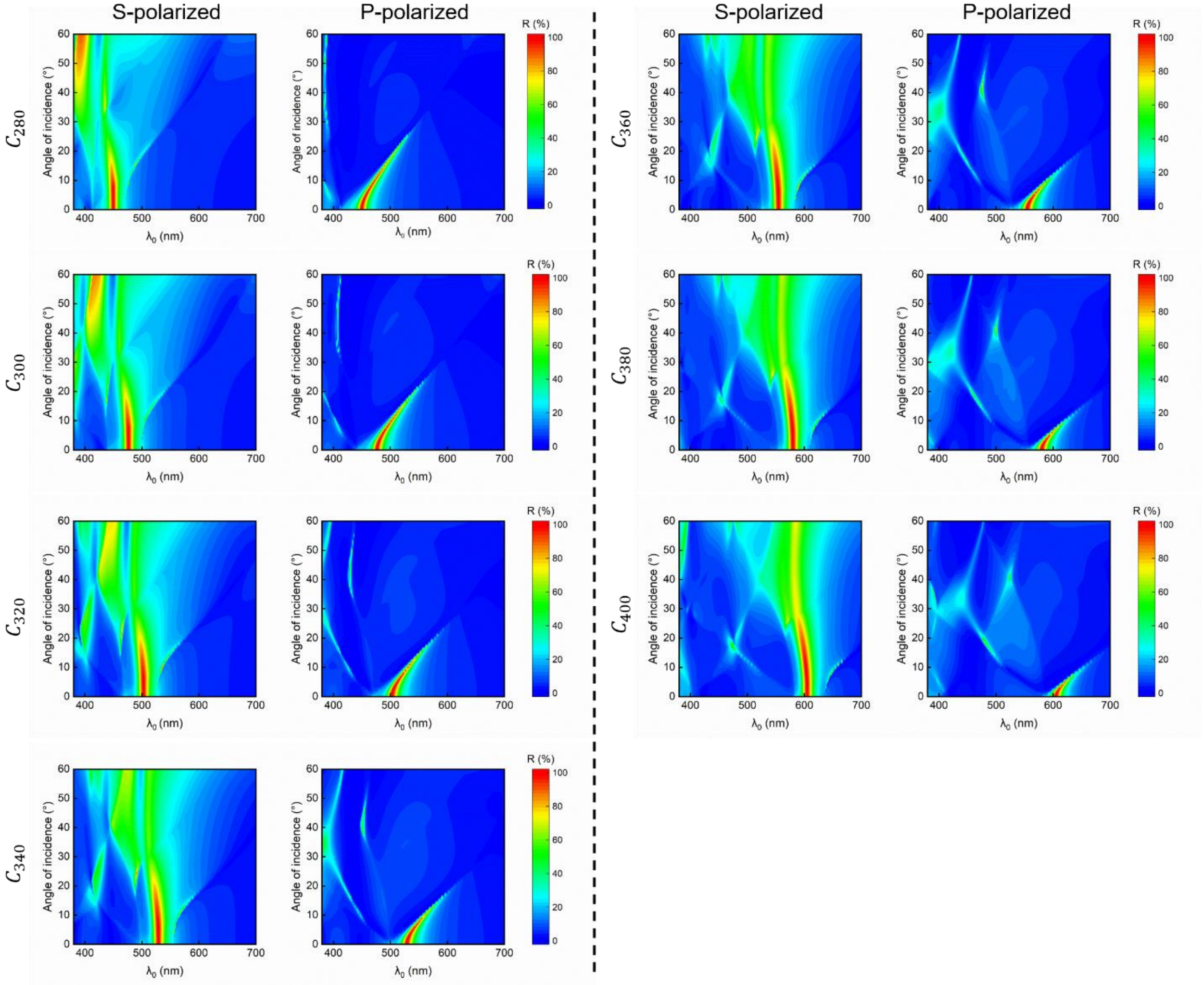

Figure S4: Angular response of the designed Ta_2O_5 structural color generating metasurfaces for incident light polarized perpendicular to the plane of incidence (s-polarized) and parallel to the plane of incidence (p-polarized).

V. Ways to tune the spectral linewidth of the Ta_2O_5 structural color generating metasurfaces

For the designed Ta_2O_5 structural color generating metasurface, its spectral linewidth can be adjusted by varying the geometric parameters of the nanopillars. Taking a green-color device for example (Fig. S5), its spectral linewidth can be tuned by varying the height of the pillar from 160 nm to 240 nm, while fixing both its period (P = 340 nm) and filling ratio ($f = D/P = 0.7$). It is worth noting that there is generally a trade-off between the color purity and color brightness. When

the spectral linewidth gets narrower (better color purity), the reflected light intensity will be reduced (lower color brightness).

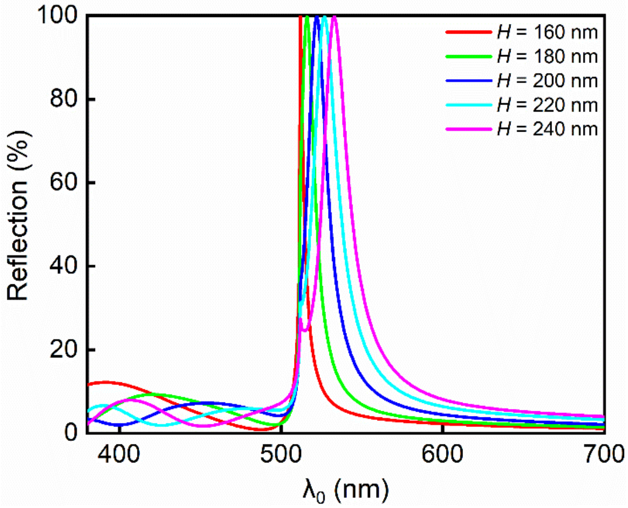

Figure S5: Simulated reflection spectra of green-color Ta_2O_5 metasurfaces, which have identical period (P = 340 nm) and filling ratio ($f = D/P = 0.7$), but different pillar heights (H).

VI. CIE 1931 XYZ chromaticity coordinates based on the simulated and measured spectra of the Ta_2O_5 structural color generating metasurfaces

We calculate the color coordinates based on both the simulated (black circles) and measured (black triangles) spectra of the Ta_2O_5 structural color generating metasurfaces, and plot the coordinates in the CIE 1931 XYZ chromaticity diagram (Fig. S6). A majority of the obtained color coordinates are located close to the diagram's outer edge, showing a good saturation level. In addition, the enclosed area of these coordinates is comparable to the triangular area enclosed by the standard RGB (sRGB) color coordinates, showing a wide color gamut coverage. To further extend the color coverage into the red-color region, we can tune the geometric parameters of the Ta_2O_5 nanopillars. The four blue circles represent the color coordinates based on the simulated spectra from Ta_2O_5 nanopillars with the geometric parameters (P, H, D) respectively as (400 nm, 260 nm, 140 nm), (400 nm, 280 nm, 140 nm), (400 nm, 240 nm, 145 nm), and (400 nm, 260 nm, 145 nm).

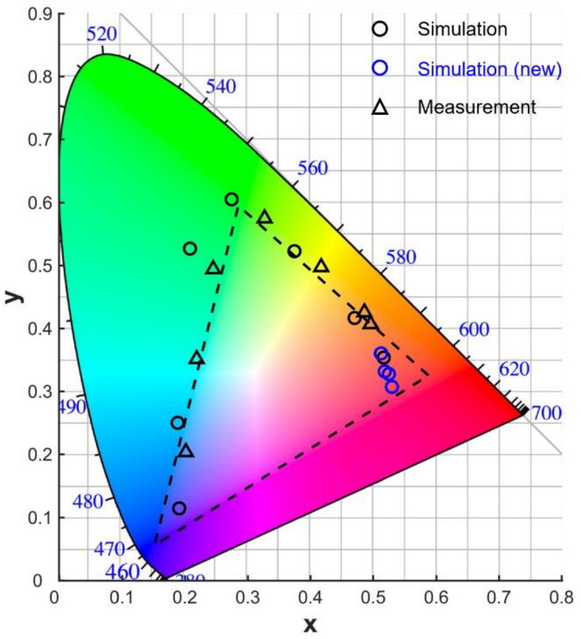

Figure S6: CIE 1931 XYZ chromaticity coordinates based on the simulated and measured spectra of the Ta_2O_5 structural color generating metasurfaces.
