# Supplementary figures and images for: Tantalum pentoxide: a new material platform for high-performance dielectric metasurface optics in the ultraviolet and visible region
Source: Light Sci Appl. 2024 Jan 22;13:23. doi: 10.1038/s41377-023-01330-z (PMC10800353; doi:10.1038/s41377-023-01330-z)

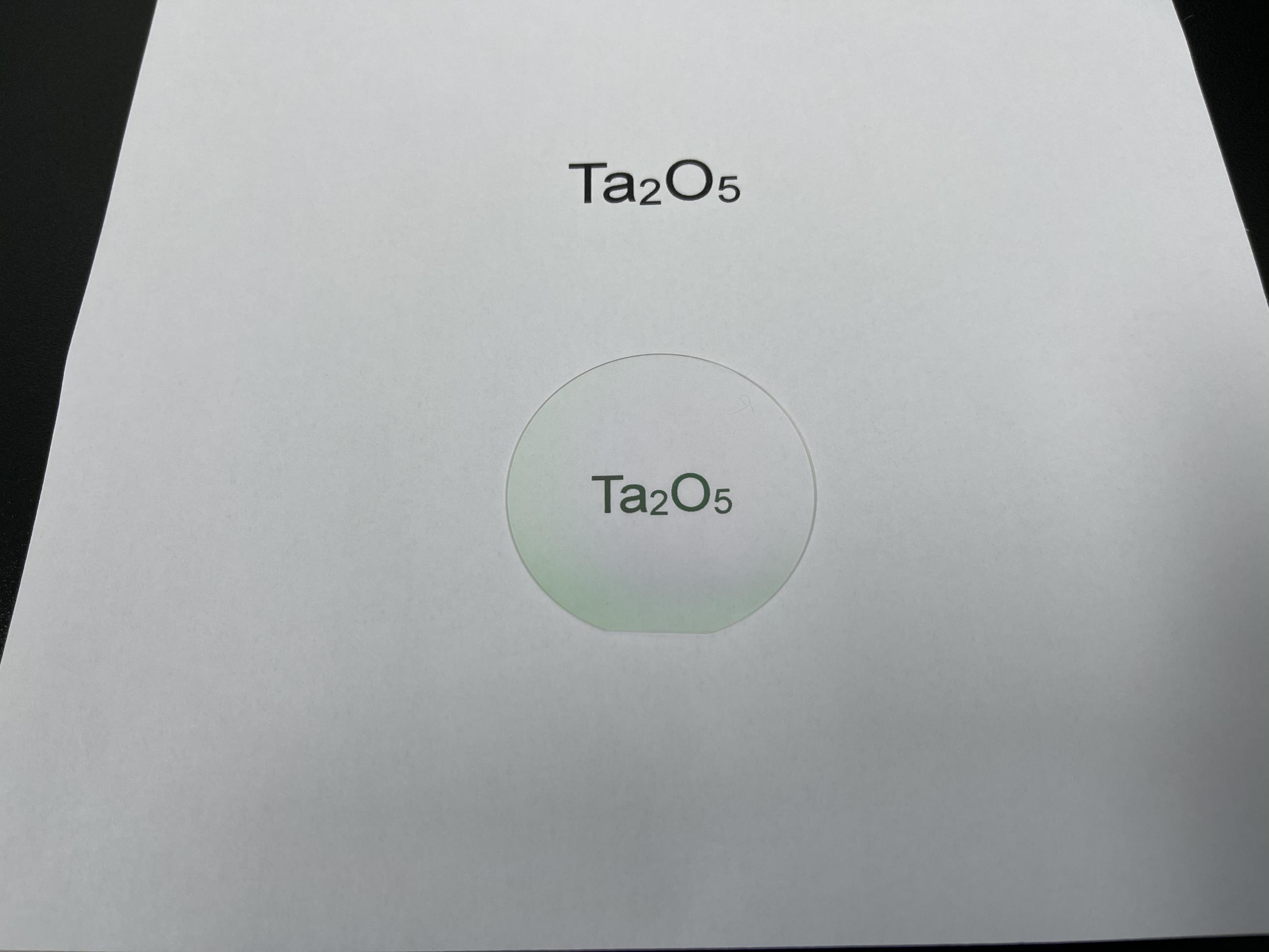

Supplement: Supplementary file 2 — Figure 1 [file 41377_2023_1330_MOESM2_ESM.zip › visio/media/image6.jpeg]

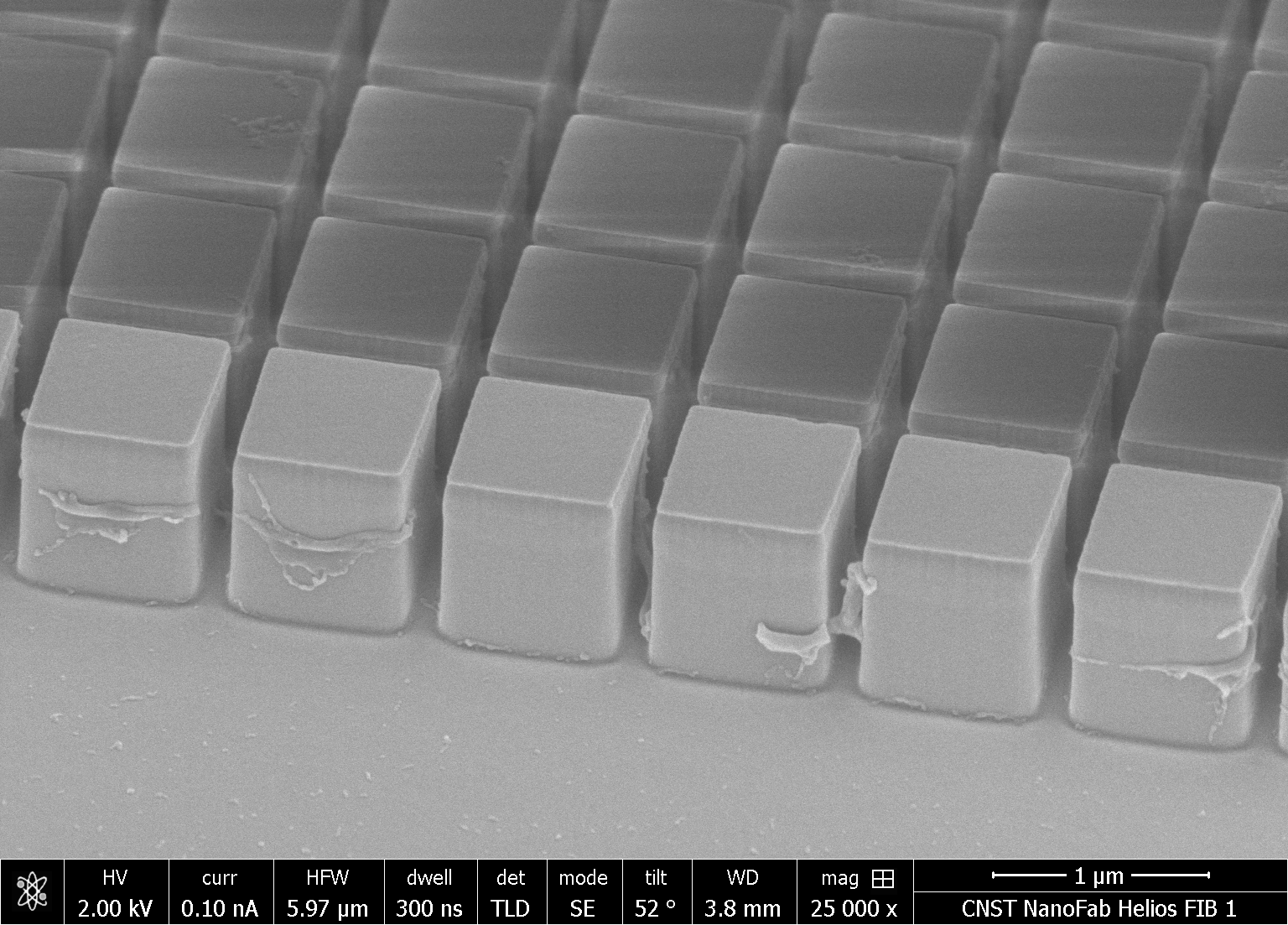

Supplement: Supplementary file 2 — Figure 1 [file 41377_2023_1330_MOESM2_ESM.zip › visio/media/image4.tiff]

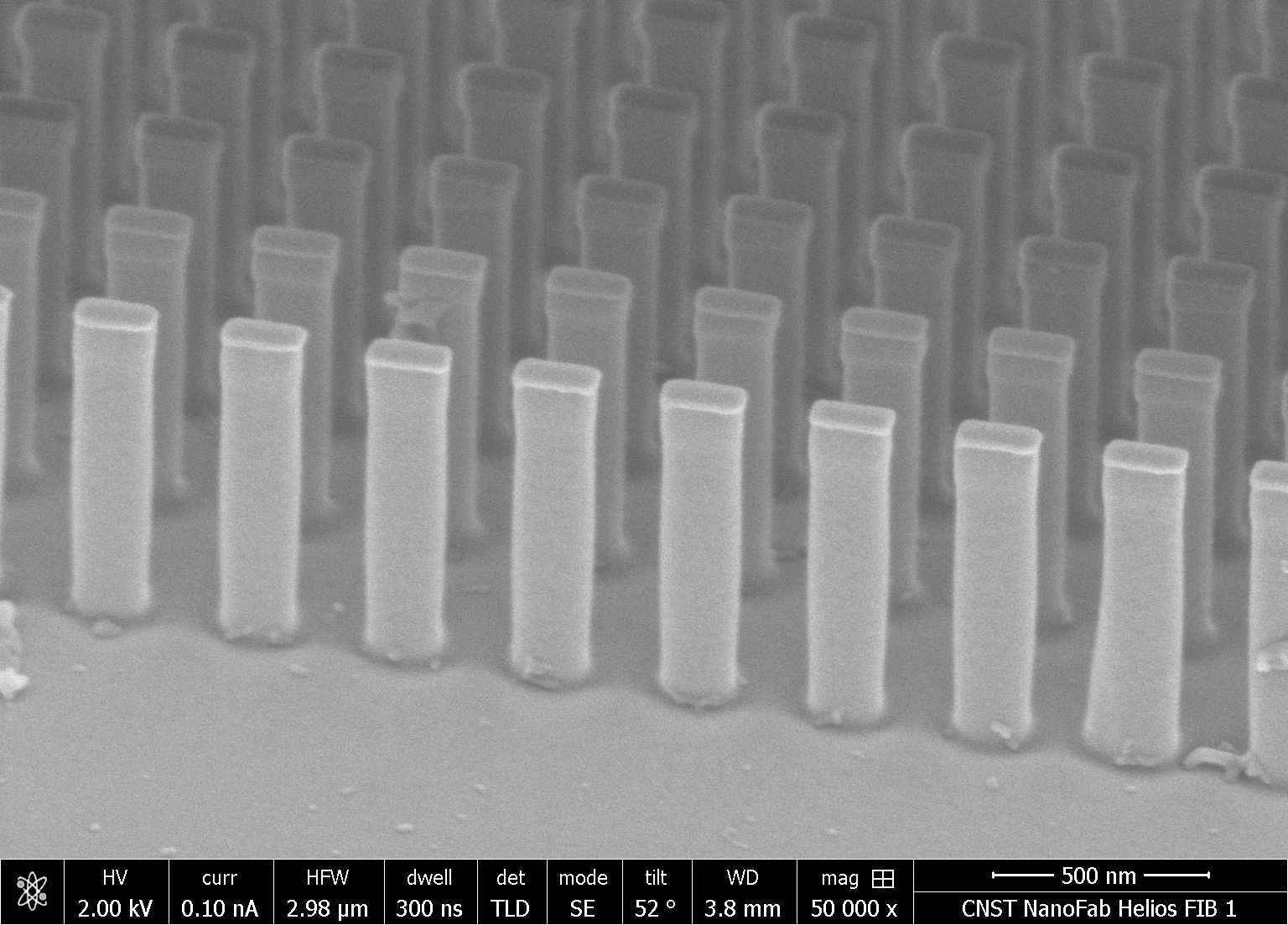

Supplement: Supplementary file 2 — Figure 1 [file 41377_2023_1330_MOESM2_ESM.zip › visio/media/image3.tiff]

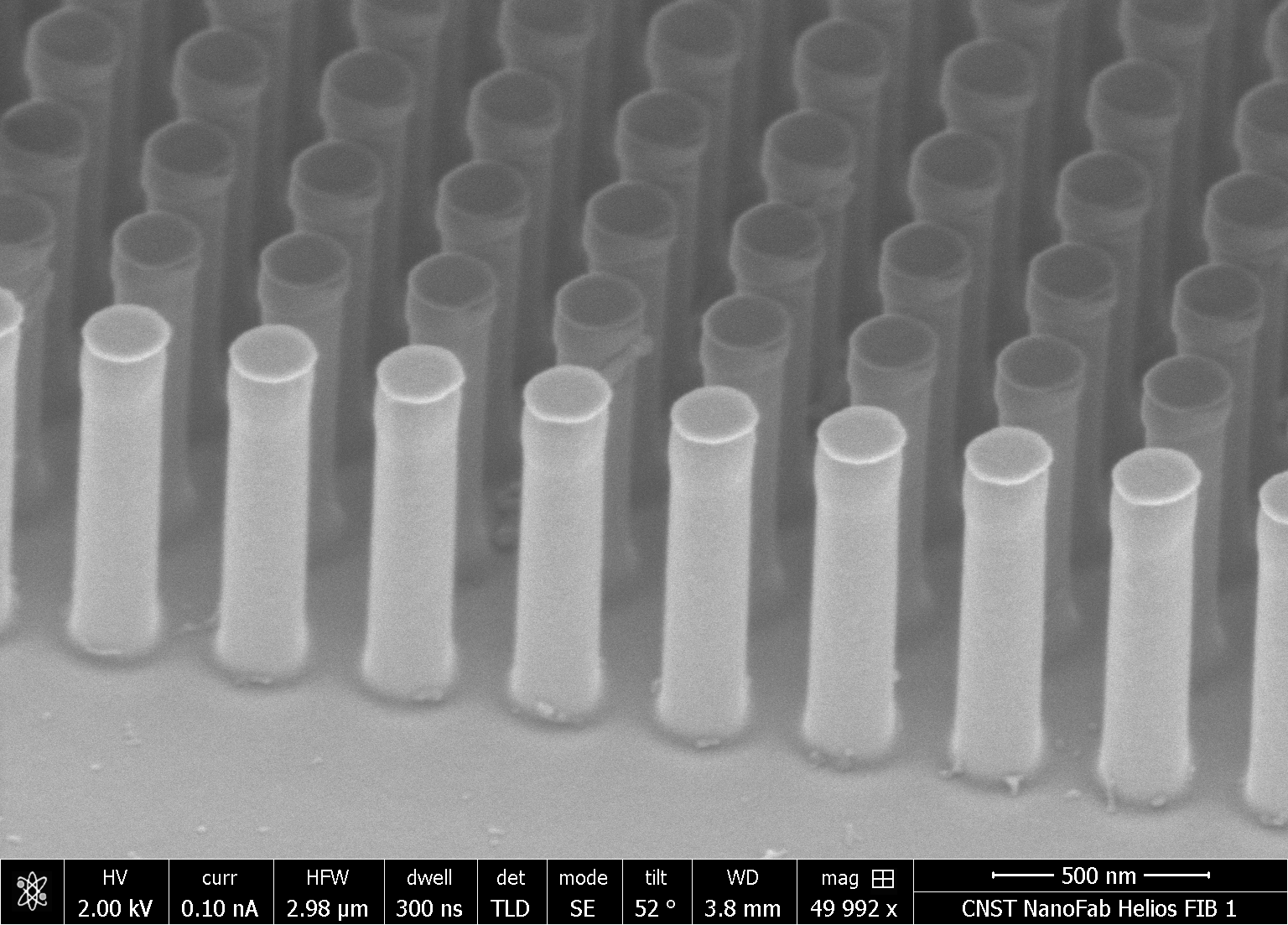

Supplement: Supplementary file 2 — Figure 1 [file 41377_2023_1330_MOESM2_ESM.zip › visio/media/image2.tiff]

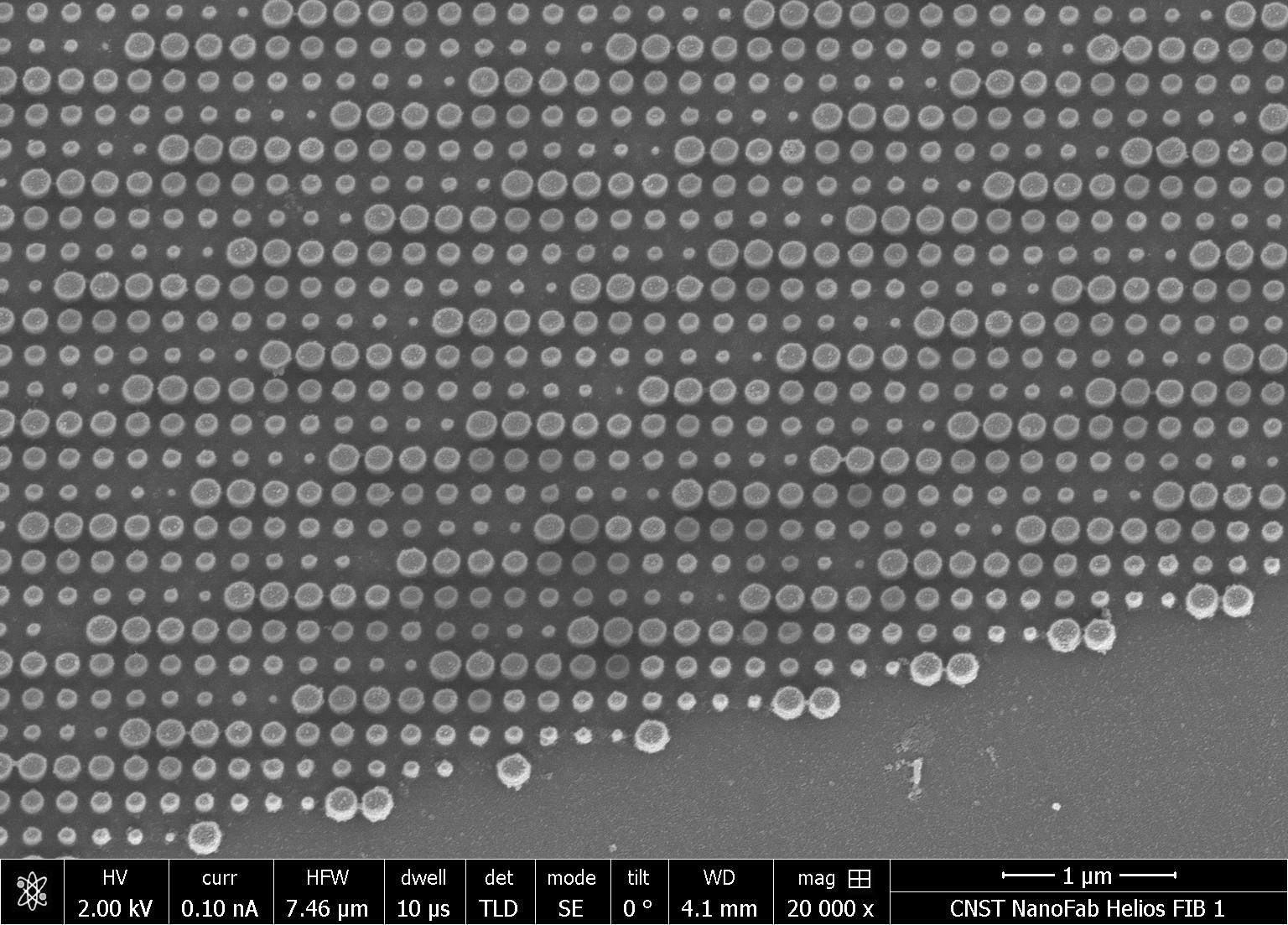

Supplement: Supplementary file 3 — Figure 2 [file 41377_2023_1330_MOESM3_ESM.zip › visio/media/image10.tiff]

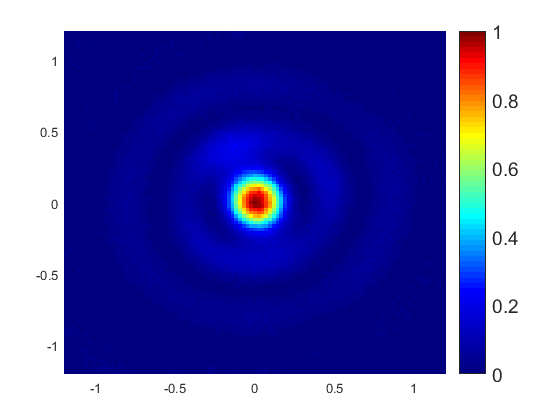

Supplement: Supplementary file 3 — Figure 2 [file 41377_2023_1330_MOESM3_ESM.zip › visio/media/image2.tiff]

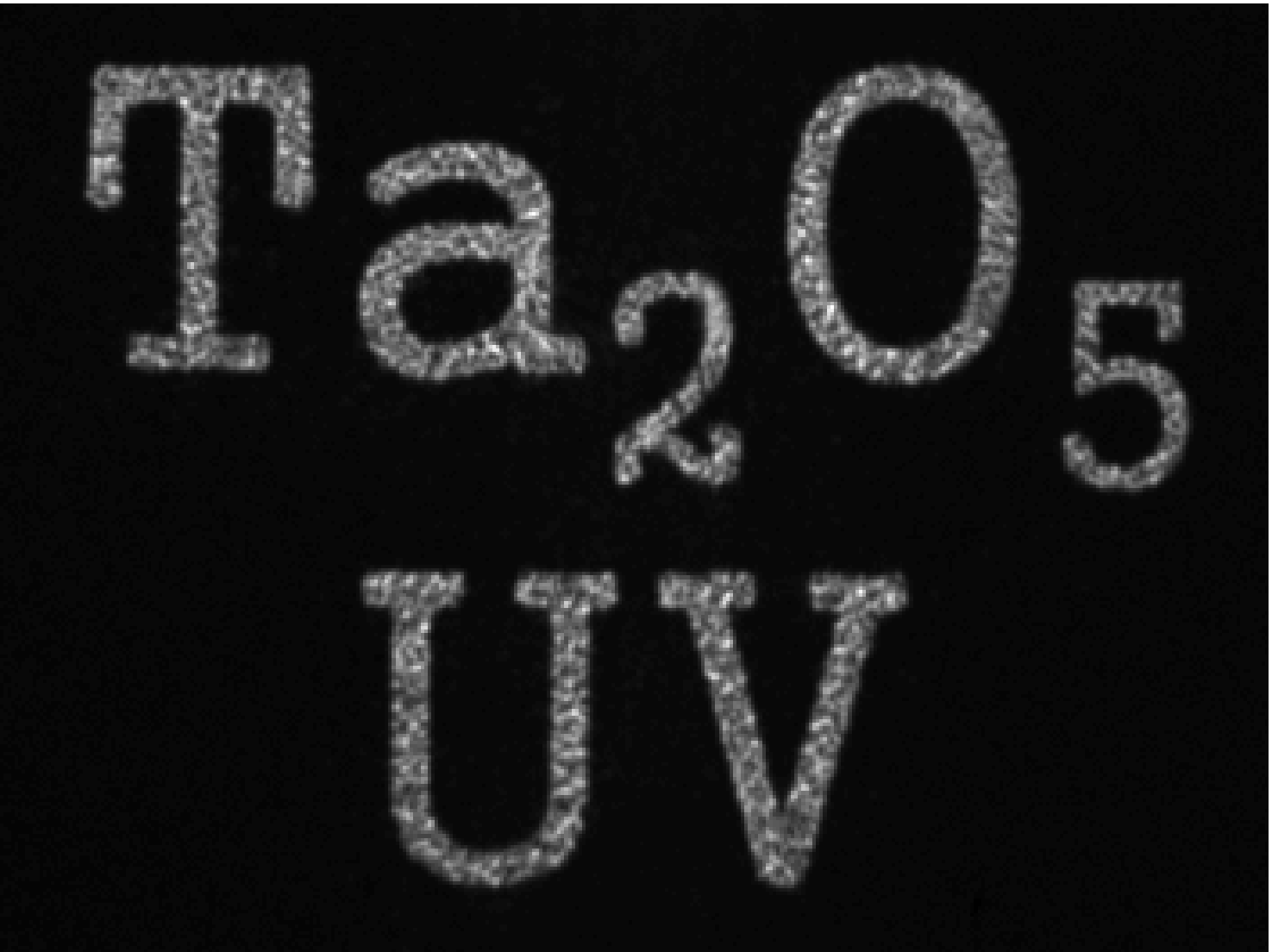

Supplement: Supplementary file 4 — Figure 3 [file 41377_2023_1330_MOESM4_ESM.zip › visio/media/image8.tiff]

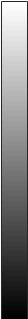

Supplement: Supplementary file 4 — Figure 3 [file 41377_2023_1330_MOESM4_ESM.zip › visio/media/image9.tiff]

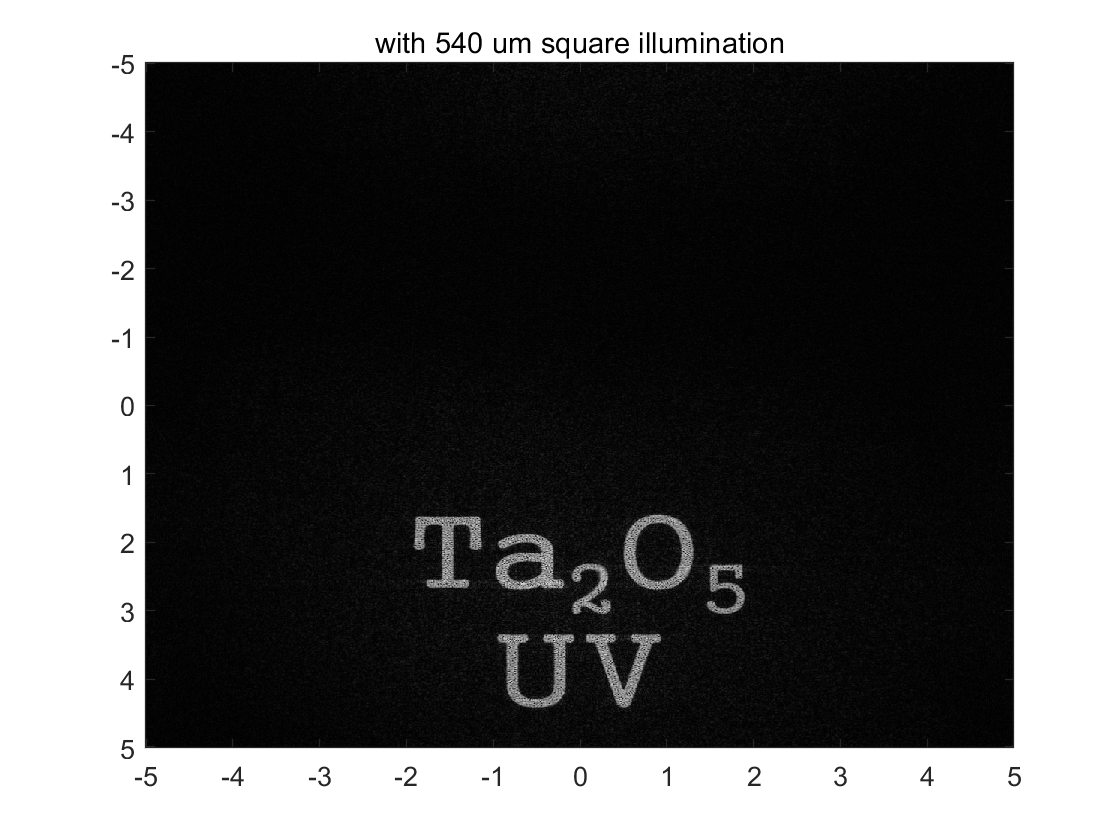

Supplement: Supplementary file 4 — Figure 3 [file 41377_2023_1330_MOESM4_ESM.zip › visio/media/image7.tiff]

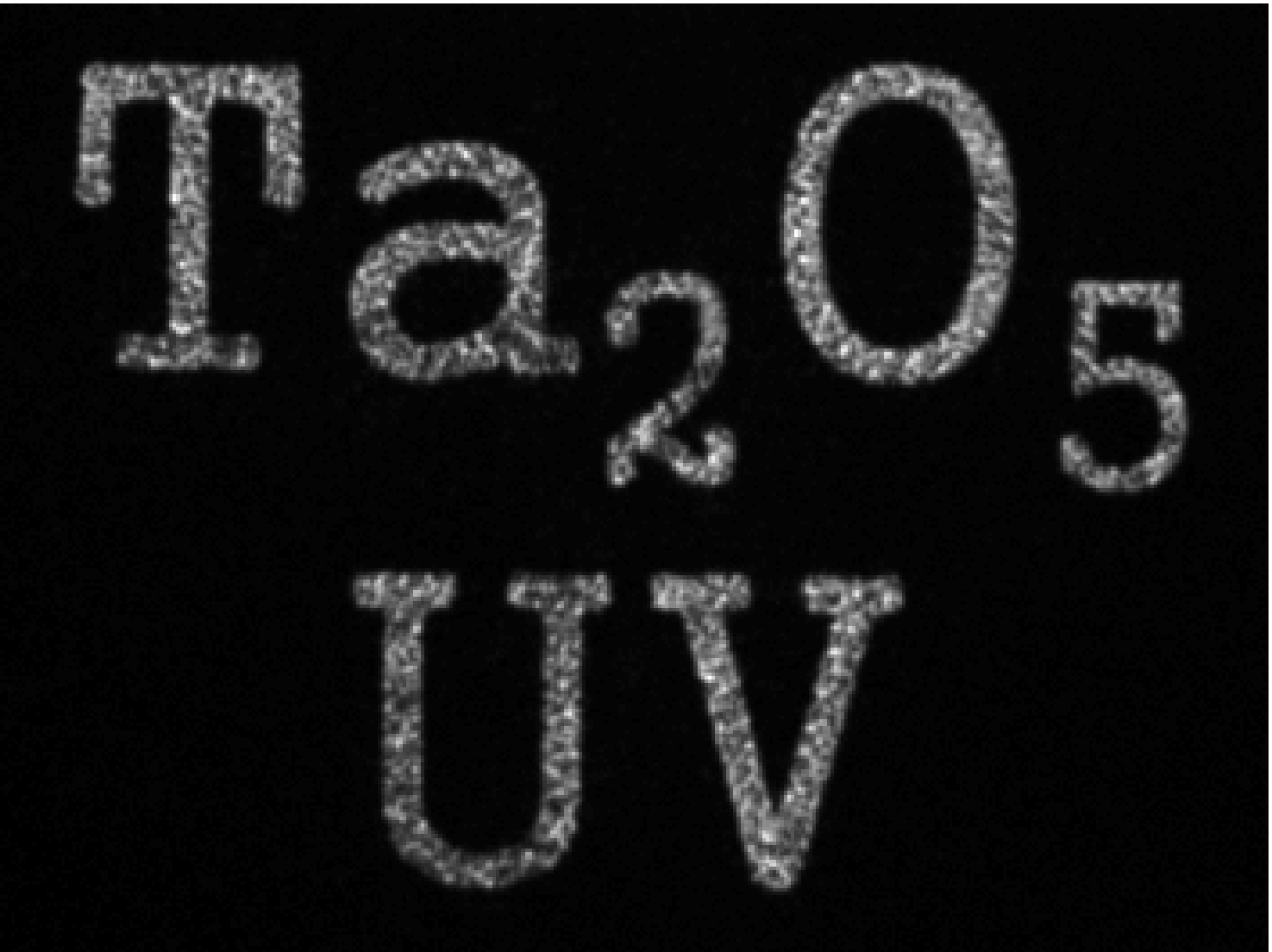

Supplement: Supplementary file 4 — Figure 3 [file 41377_2023_1330_MOESM4_ESM.zip › visio/media/image6.tiff]

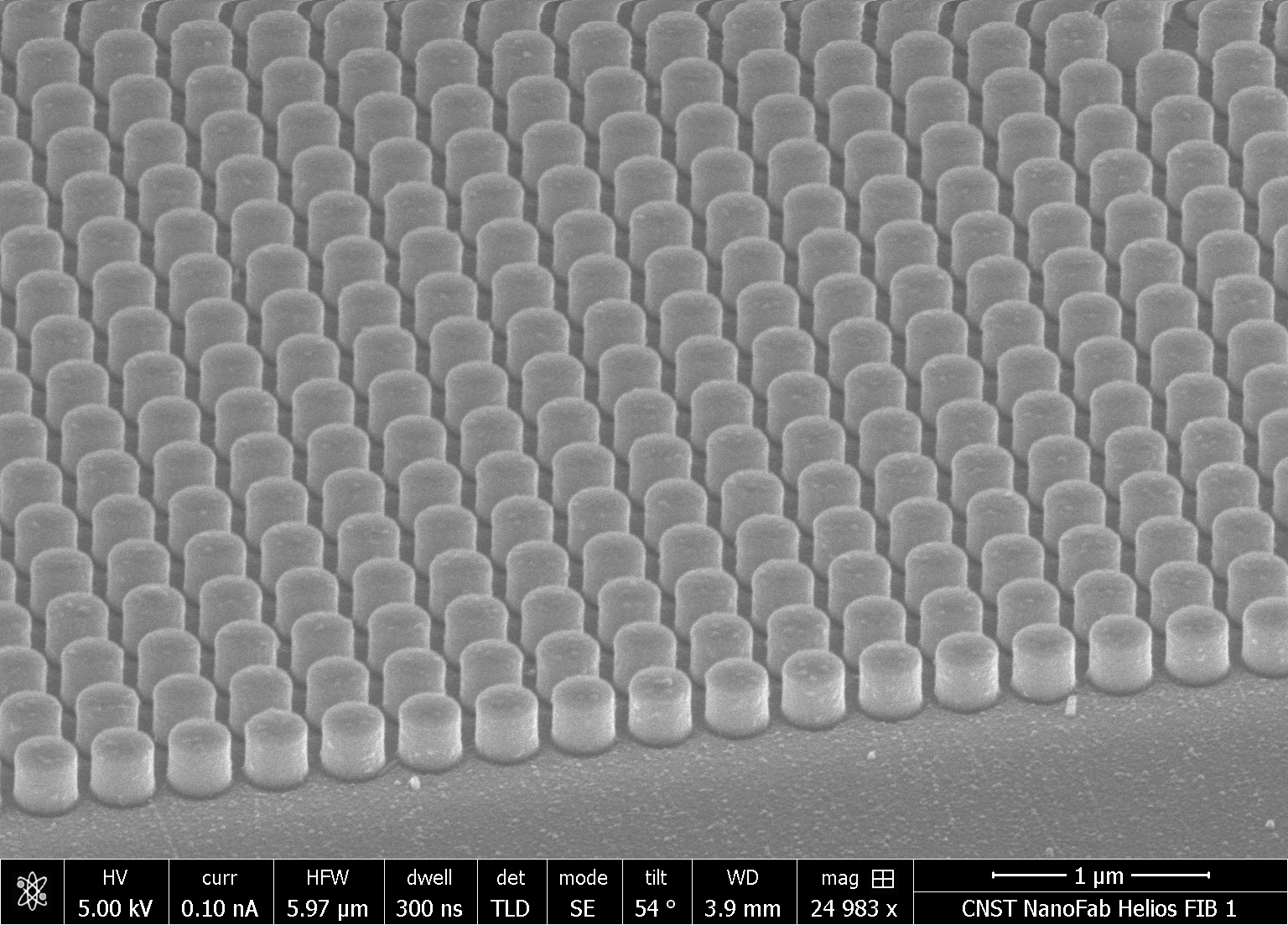

Supplement: Supplementary file 5 — Figure 4 [file 41377_2023_1330_MOESM5_ESM.zip › visio/media/image9.tiff]

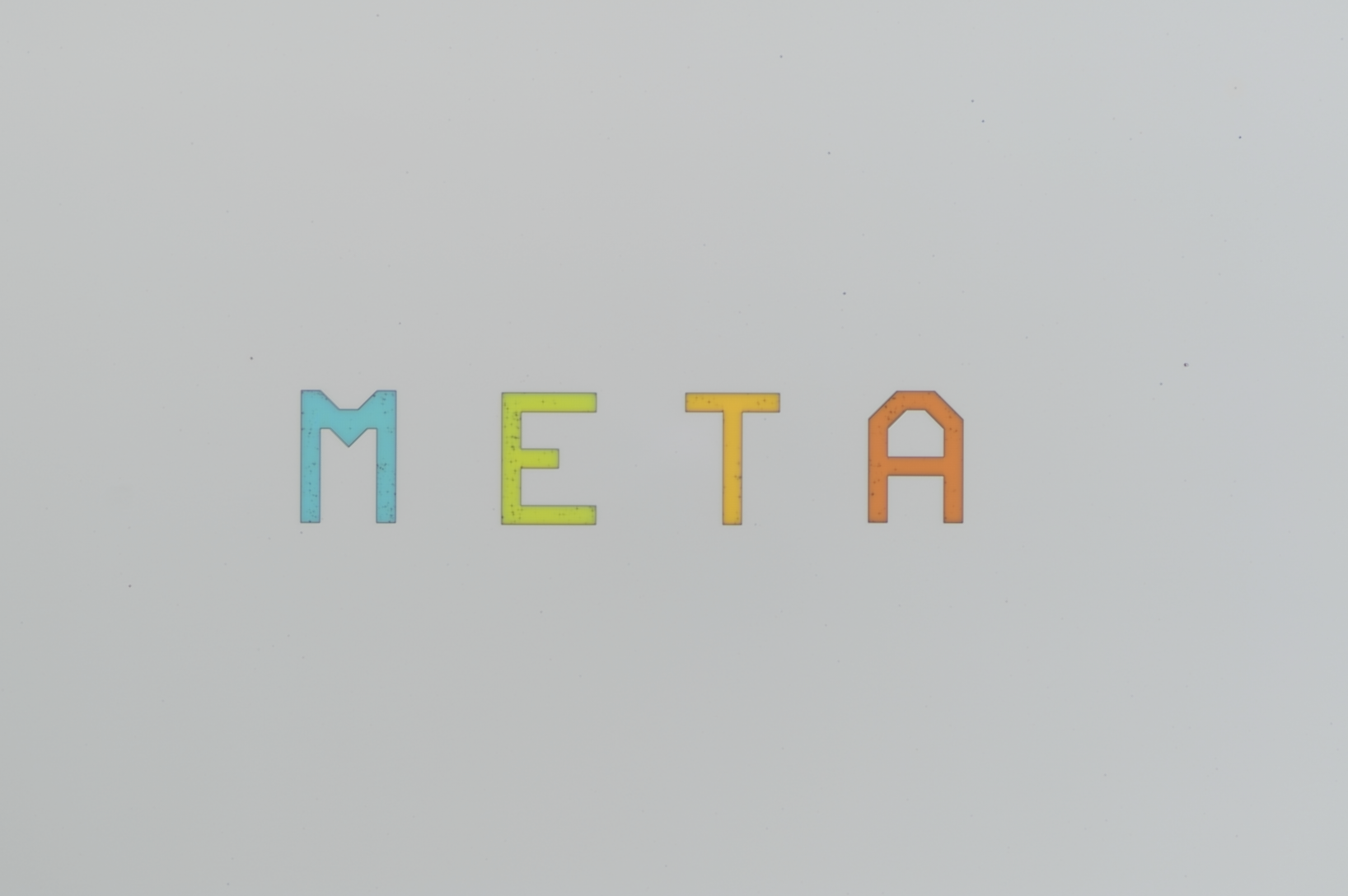

Supplement: Supplementary file 5 — Figure 4 [file 41377_2023_1330_MOESM5_ESM.zip › visio/media/image2.tiff]

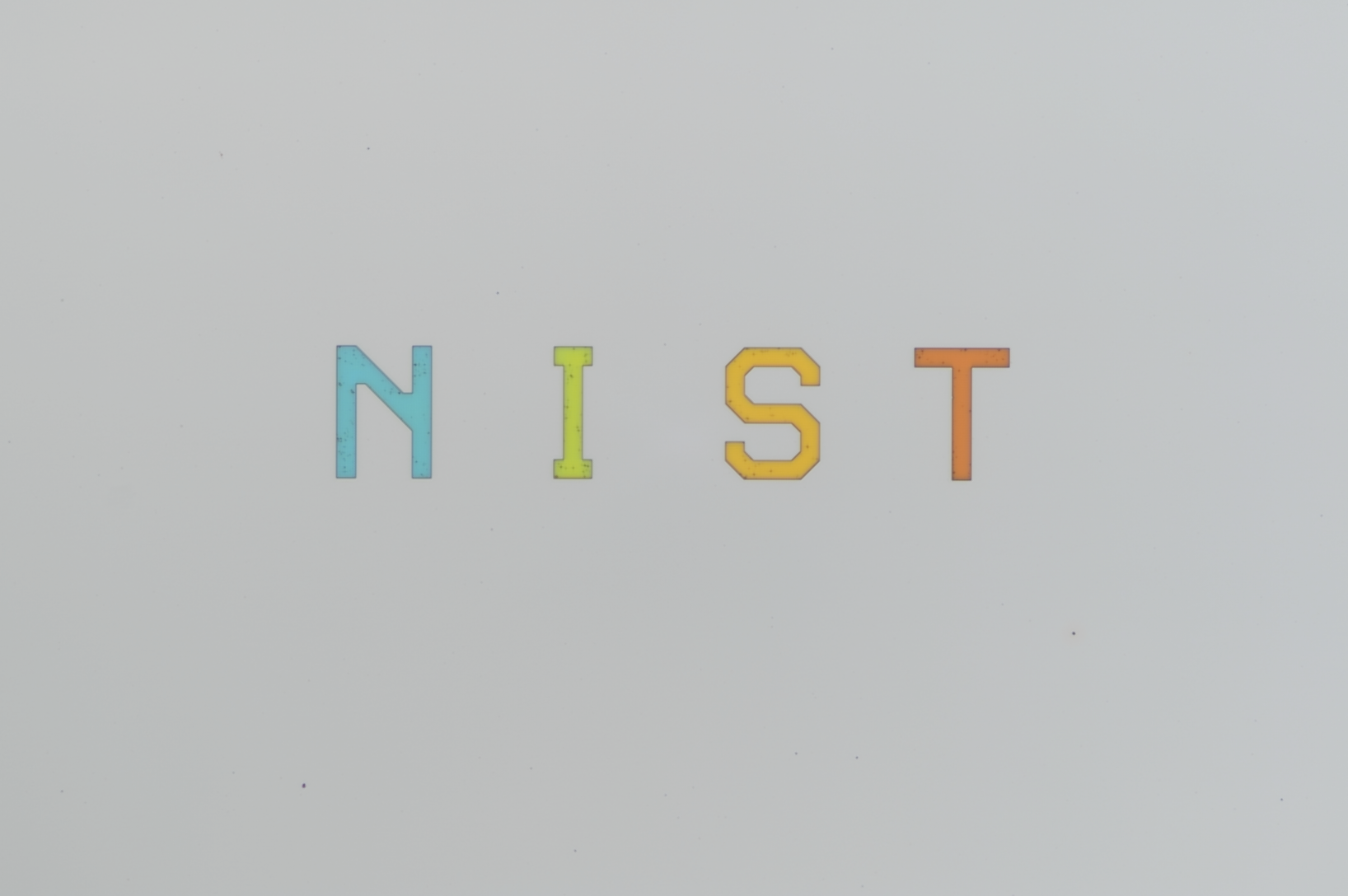

Supplement: Supplementary file 5 — Figure 4 [file 41377_2023_1330_MOESM5_ESM.zip › visio/media/image3.tiff]
